# Non-Destructive Monitoring of Crop Fresh Weight and Leaf Area with a Simple Formula and a Convolutional Neural Network

**DOI:** 10.3390/s22207728

**Published:** 2022-10-12

**Authors:** Taewon Moon, Dongpil Kim, Sungmin Kwon, Tae In Ahn, Jung Eek Son

**Affiliations:** 1 Research Institute of Agriculture and Life Sciences, Seoul National University, Seoul 08826, Korea; 2 Department of Agriculture, Forestry and Bioresources, Seoul National University, Seoul 08826, Korea

**Keywords:** artificial intelligence, deep learning, machine learning, plant environment, precision agriculture

## Abstract

Crop fresh weight and leaf area are considered non-destructive growth factors due to their direct relation to vegetative growth and carbon assimilation. Several methods to measure these parameters have been introduced; however, measuring these parameters using the existing methods can be difficult. Therefore, a non-destructive measurement method with high versatility is essential. The objective of this study was to establish a non-destructive monitoring system for estimating the fresh weight and leaf area of trellised crops. The data were collected from a greenhouse with sweet peppers (*Capsicum annuum* var. *annuum*); the target growth factors were the crop fresh weight and leaf area. The crop fresh weight was estimated based on the total system weight and volumetric water content using a simple formula. The leaf area was estimated using top-view images of the crops and a convolutional neural network (ConvNet). The estimated crop fresh weight and leaf area exhibited average R^2^ values of 0.70 and 0.95, respectively. The simple calculation was able to avoid overfitting with fewer limitations compared with the previous study. ConvNet was able to analyze raw images and evaluate the leaf area without additional sensors and features. As the simple calculation and ConvNet could adequately estimate the target growth factors, the monitoring system can be used for data collection in practice owing to its versatility. Therefore, the proposed monitoring system can be widely applied for diverse data analyses.

## 1. Introduction

Adequate crop growth leads to high productivity. Crop growth responses to the environment and the growth rate of each organ can differ [1,2]. For efficient cultivation management, crop growth should be quantified; however, it is difficult to measure the factors that directly determine crop growth. Therefore, relevant factors should be used to estimate crop growth, and adequate growth factors should be selected.

Fresh weight is directly related to the productivity of leafy vegetables; for fruit vegetables, it can be used as an indicator for determining the reproductive stage or the growth rate [3,4,5]. Several measuring systems have been developed to directly measure the crop fresh weight as a growth-related factor [6,7,8].

However, it is difficult to measure the fresh weight of trellised crops with high-wires in greenhouses due to the substrate and water weight [9]. In this case, the total weight, including the substrate and water weight, is measured for crop management [7,8,10]. The crop fresh weight can be estimated using some assumptions and formulas; however, the data and features related to substrates and crops should be re-investigated under different cultivation conditions for parameter calibration. Therefore, a convenient approach requiring fewer parameters is essential to monitor the crop fresh weight and increase the versatility of the measuring system.

Another growth-related factor is the leaf area or leaf area index. It can be used to determine plant light interception and photosynthesis [11,12,13]. Based on its relationship with carbon assimilation, diverse measuring methodologies for several plants have been introduced [14,15,16]. The leaf area can be directly measured because all leaves are exposed, but the direct measurement method is not practical for agricultural systems larger than the farm scale. Instead, the leaf area index of sweet peppers was estimated based on optical simulations and 3D-scanned plants [17]; however, this estimation process hindered the reusability of the method. Therefore, an indirect measurement method with high versatility is essential.

Computer vision is widely used as an indirect measurement method for diverse purposes in agriculture [18,19]. Recently, with technological advances, raw-state images obtained using aerial photographs and commercial cameras have been utilized for measurement in various agricultural systems, such as fields and greenhouses [20,21]. Growth factors were also estimated for leafy vegetables using raw data from top-view images and computer vision [22]. However, for trellised crops, the distance between target crops and cameras tends to reduce because of the increasing crop height. Therefore, the applicability of a similar method should be verified under on-the-ground conditions such as greenhouses.

In addition to computer vision, deep learning has been used in various fields due to its flexibility [23]. Deep neural networks (DNNs), a core part of deep learning algorithms, are also actively adapted for horticulture fields that require distinguishable domain knowledge [24]. Therefore, adequately trained DNNs can convert raw data into selected targets, such as crop growth factors, without delicate calibration. The objective of this study was to establish a growth monitoring system for trellised crops that can easily estimate the fresh weight and leaf area of crops with daily data. Weight can be measured only with scales, and leaf area can be directly estimated with images. We attempted to improve the measuring device and algorithms in terms of the versatility of the developed system.

## 2. Materials and Methods

### 2.1. Cultivation Conditions

**Figure 1:**
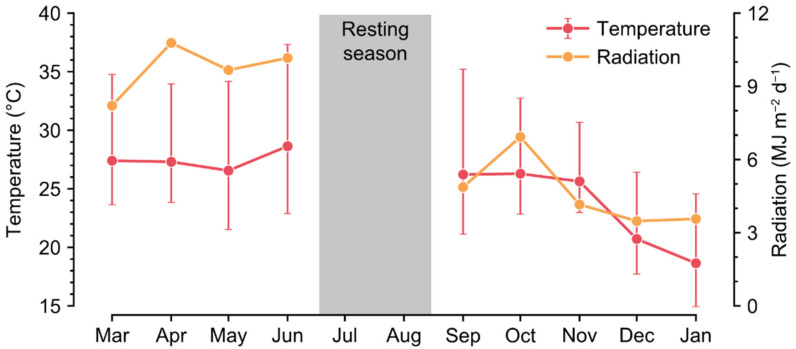
Daily temperature and radiation in the greenhouse. Minimum and maximum values for temperature are shown. No cultivation was conducted in the resting season. The crops were transplanted and grown from 26 February 2020 to 3 July 2020 (2020S), and from 25 August 2020 to 24 January 2021 (2020W).

Sweet peppers (*Capsicum annuum* var. *annuum*) were cultivated in a Venlo-type greenhouse at the experimental farm of Seoul National University, Suwon, Korea (37.3° N, 127.0° E). The crops were transplanted and grown from 26 February 2020 to 3 July 2020 (2020S) and from 25 August 2020 to 24 January 2021 (2020W). Cultivation details, such as cultivar and planting density, were differed with each period for conditional variation. cv. Scirocco and cv. Mavera were cultivated in 2020S and 2020W, respectively. A stone wool slab and cubes (Grodan GT Master, Grodan, Roermond, The Netherlands) were used as substrates. Four and three crops were transplanted for each substrate in 2020S and 2020W, respectively (Table 1). The two main stems of the crops were maintained with trellis strings. The crops were grown in four rows, and the number of slabs per row was seven. In 2020W, shoot apical meristems were eliminated to prevent unnecessary vegetative growth on 5 December 2020 (103 days after transplanting, DAT). Daytime and nighttime temperatures for the environment controller were set at 25–35 °C and 17–22 °C, respectively (Figure 1). The nutrient composition was based on the PBG nutrient solution from the Netherlands. Electrical conductivity (EC) of nutrient solutions was maintained between 2.8–3.2 dS m^−1^. An integrated solar radiation method was applied for irrigation control (0.5 MJ m^−2^ of the accumulated solar radiation, 66 mL per dripper). The fruits were harvested three times a week when the surfaces of the fruits were mostly colored.

### 2.2. Data Collection

Greenhouse environmental data were measured every ten minutes. Temperature and relative humidity were measured using a complex sensor (AQ3020, Aosong Electronics, Guangzhou, China), and radiation was measured using a pyranometer (SQ-110, Apogee Instrument Inc., Logan, UT, USA). The rhizosphere environment was also measured using environmental data (TEROS 12, Meter Group Inc., Pullman, WA, USA).

**Figure 2:**
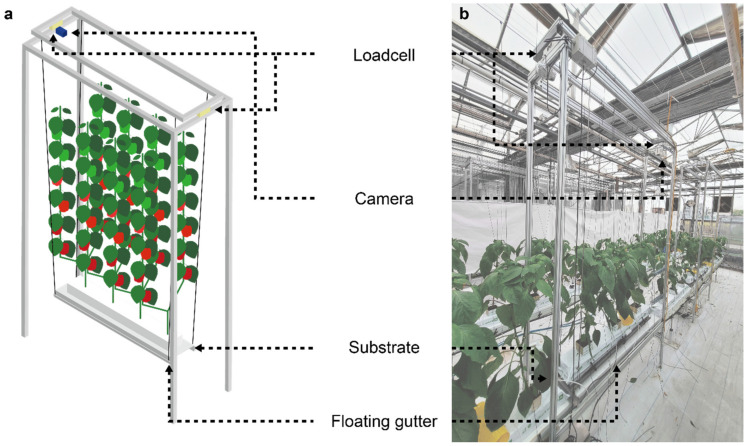
Structure of the monitoring system. (**a**) Mimetic diagram; (**b**) photograph of the real device. A substrate moisture sensor was directly installed upon a floating gutter, minimizing interruption.

The fresh weight measuring device developed by Lee & Son (2019) [8] was modified into a crop growth monitoring system (Figure 2). The monitoring system comprised an inner frame and an outer frame. The inner frame contained crops with a substrate on a floating gutter, and the outer frame held the crops up. Two single-point load cells (CBCA-25, CURIOTEC Co., Paju, Korea) were installed to measure the total system weight including the weight of water, substrate, and crops. The scale was set to zero in the inner frame without the crops and their substrate. A camera (Dafang, Xiaomi, Beijing, China) was installed at the top of the outer frame to collect the upper crop images; the images were captured every ten minutes. In this study, three monitoring systems were installed in the middle of the cultivation area. The camera could observe three to five plants at the same time according to the development stage.

The images were collected at 15–125 and 17–102 DAT for 2020S and 2020W, respectively. Images measured from 0800 to 1600 h were used as the inputs. The images were cropped, resized, and augmented for the data preprocessing (Supplementary Figure S1). The cropping was to cut 80 and 300 pixels equally for horizontal and vertical margins, respectively. Then, the cropped images were resized to 128 × 128 resolution as input, which is the size generally used for small computations. The input images were augmented by flipping, shifting, and rotating during the model training. The other data were collected at 9–128 and 24–152 DAT for 2020S and 2020W, respectively. The collected data were saved on a cloud platform (ioCrops Cloud, ioCrops Inc., Seoul, Korea). As the data were uploaded through wireless communication, small losses occurred. The missing data were interpolated using U-Net and linear interpolation [25].

The actual data for training deep learning models and validating developed methodologies were collected with destructive investigation. All the necessary samples were dried for 72 h at 80 °C in a forced-air drying oven (HB-503LF, Hanbaek Co. Ltd., Bucheon-si, Gyeonggi-do, Korea). Total destructive investigations were conducted five times for both cultivations. Four and six plants were sampled four times, and fifteen and twenty plants were sampled in the last investigations in 2020S and 2020W, respectively. The outliers were eliminated for the experiments; therefore, the total number of used samples were 63 out of 75. The substrates were investigated only at the end of each cultivation. The numbers of sampled substrates were 11 and 22 for 2020S and 2020W, respectively.

### 2.3. Calculating and Estimating Crop Fresh Weight from the Collected Data

In this study, the crop fresh weight was calculated by subtracting the substrate and water weights from the system weight. The water weight of the substrate was indirectly estimated using the volumetric water content (VWC) and substrate volume (Equations (1) and (2)).

Crop fresh weight = System weight − (Substrate weight + Water weight) (1)

Water weight = VWC × Substrate volume (2)

**Figure 3:**
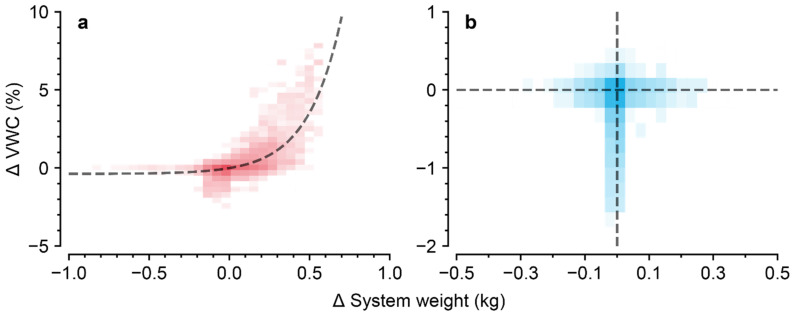
Relationship between total weight change and volumetric water content (VWC) change during (**a**) the daytime and (**b**) the nighttime. Averages of the total weight and VWC for every ten minutes, and the subtracted differences, are depicted.

Since the change in system weight was not related to the night VWC, the daily averages of the system weight and night VWC were used to exclude the water weight (Figure 3). The results obtained using the previous method developed by Lee & Son (2019) were adapted for comparison. For reproducibility, the data collection time for calculation was set to be the same. The calibration parameters for the VWC (Cf) and water weight were calculated from the data.

As deep learning approaches require no parameters except those used to model architectures, methods such as long short-term memory (LSTM), convolutional neural network (ConvNet), and Transformer have been applied to simplify the calculation [26,27,28]. In this study, the encoder structure of the Transformer was combined with a ConvNet-like decoder. The deep learning models were compared with multivariate linear regression. For the impartial comparison with the simple calculation, the input and the output were set to the daily system weight at 10 min intervals and the calculated daily crop weight, respectively.

The calculated and estimated fresh weights were also compared with the actual fresh weight collected from the destructive investigation. Since root dry weight is difficult to separate from the substrate, the substrate with roots was completely dried during the last destructive investigation (Table 2). The root dry weight was obtained by subtracting the dry weight of the empty substrate from the weight of the substrate with roots. Root dry weights on the other days were estimated using the ratio of root and shoot dry weights (RS ratio). Subsequently, the root fresh weight was estimated from the total fresh weight and the ratio of root fresh and dry weights (DF ratio); the DF ratio was consistent with that obtained in the previous study [8]. The estimated root fresh weight was added to the fresh weight data from the destructive investigation.

### 2.4. Estimation of Leaf Area Using a ConvNet

As the daily leaf area was estimated from images, only a 2D ConvNet was used for the estimation. The ConvNet, one of the deep learning algorithms, consists of several convolution layers. The convolution process helps the network to abstract the given input as a desired output [29]. The ConvNet’s algorithms yield state-of-the-art performances in image processing based on its automated high-level abstraction [27,30,31]. Therefore, the ConvNet was used to increase the applicability of leaf area estimation.

Leaf area as label data for model training was collected from the destructive investigation and image analysis [32]. Since the measured data for leaf area could not cover all the cultivation periods, the leaf area values were regressed to *DAT* with a sigmoidal function with arbitrary coefficients *L*, *k*, *x*_0_, and *b* (Equation (3)).

Leaf area = *L*/*exp*(*k*(*DAT* + *x*_0_) + *b*) (3)

Outliers were eliminated from the regression. The regressed values were set to the output of the ConvNet instead of the actual values from the destructive investigations. The input of the ConvNet was ten-min-interval images, and the output was the daily leaf area. Therefore, images from the same date were assigned the same label. At the model test, the output of the trained ConvNet at the same date was averaged, and the value was compared with the regressed daily leaf area.

### 2.5. Deep Learning Computation

AdamOptimizer was used for model training [33]. The models were trained to minimize the mean absolute error (MAE). Batch and layer normalizations were used for regularization [34,35]. The models were evaluated based on R2 and root mean square error (RMSE). The model structures and hyperparameters were empirically optimized (Supplementary Tables S1 and S2). TensorFlow software (v. 2.6.0, Google Inc., Mountain View, CA, USA) was used to build the model [36]. All computations were conducted using a Linux server with one GPU with 35.58 TFlops (RTX 3090, NVIDIA, Santa Clara, CA, USA).

### 2.6. Evaluation of the Monitoring System

**Figure 4:**
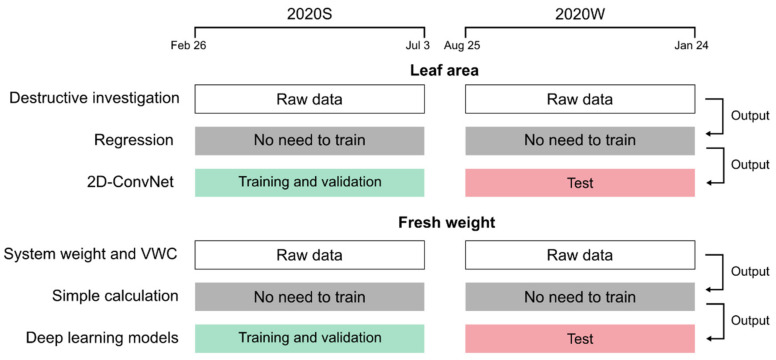
Data division for the model training. Training and validation data were randomly divided at the ratio of 7:3. The total number of data points and images were 363 and 6392, respectively. The regression of leaf area and the calculation of the fresh weight did not require the model training. The regressed leaf area was the output of the 2D ConvNet; and the calculated fresh weight was the output of the deep learning models estimating fresh weight.

Crop growth factors must be identified within cultivation periods to implement the monitoring system effectively. In this study, it was assumed that the monitoring system estimated the daily fresh weights and leaf areas at the end of the day when the daily data were collected. Thus, the estimation should be performed for different cultivation periods. The deep learning models required separated data for the model training, unlike the regression and the calculation (Figure 4).

Therefore, the models were trained with the data of 2020S, and the trained models were tested with 2020W data, with different climates and cultivational conditions. The data of 2020S were randomly divided into training and validation sets; the ratio of the training and validation sets was 7:3.

## 3. Results

### 3.1. Calculation of Crop Fresh Weight

**Figure 5:**
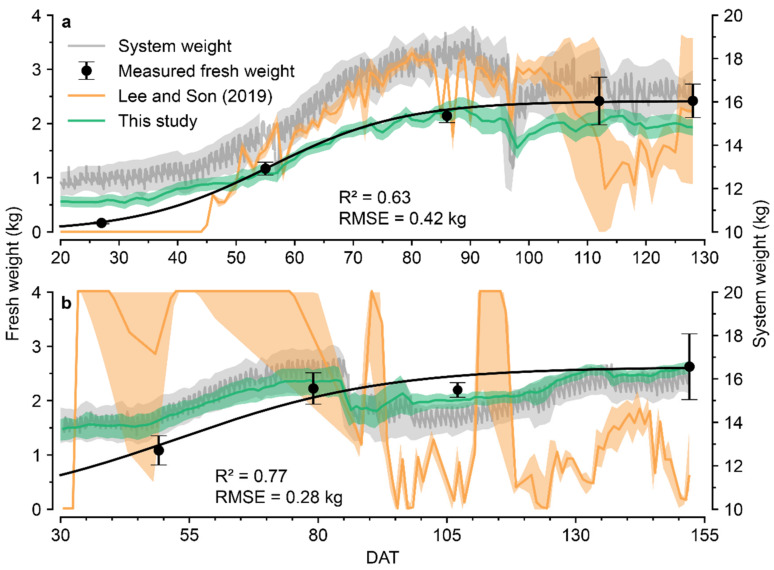
Calculated crop fresh weights in this study were obtained through the simple calculation and the method in the previous study by Lee & Son (2019). System weight, measured fresh weight, and calculated fresh weights (**a**) from 26 February 2020 to 3 July 2020 (2020S) and (**b**) from 25 August 2020 to 24 January 2021 (2020W) are depicted. The system weight represents the total weight measured from the device. The standard deviation of the day is represented by a shaded area. R^2^ and RMSE were obtained from the simple calculation. The values outside the range 0–4 kg were adjusted for legibility.

The calculated fresh weight fluctuated with increasing DAT and showed average R^2^ = 0.63 and 0.77 for 2020S and 2020W, respectively (Figure 5). In 2020S, the system weight plunged at 95 DAT, but the decrease in the calculated fresh weight was relatively moderate. Similarly, in 2020W, the calculated fresh weight could better reflect the decrease in fresh weight resulting from hard pruning and harvest compared with the regression (Equations (1) and (2)). In the early cultivation period, both calculations overestimated the measured fresh weight; in the latter part, the calculation moderately underestimated the target in 2020S. However, the results obtained using the calculation method did not deviate from the general tendency of the fresh weight changes. In contrast, Lee & Son (2019) could not accurately calculate the fresh crop weight. Their calculation reflected the measured fresh weight in 2020S, but the results of 2020W did not reflect the changing tendency accurately. The basic methodology that converts VWC to water weight was the same, but with different calculations.

### 3.2. Estimation Accuracy for the Calculated Fresh Weight

**Figure 6:**
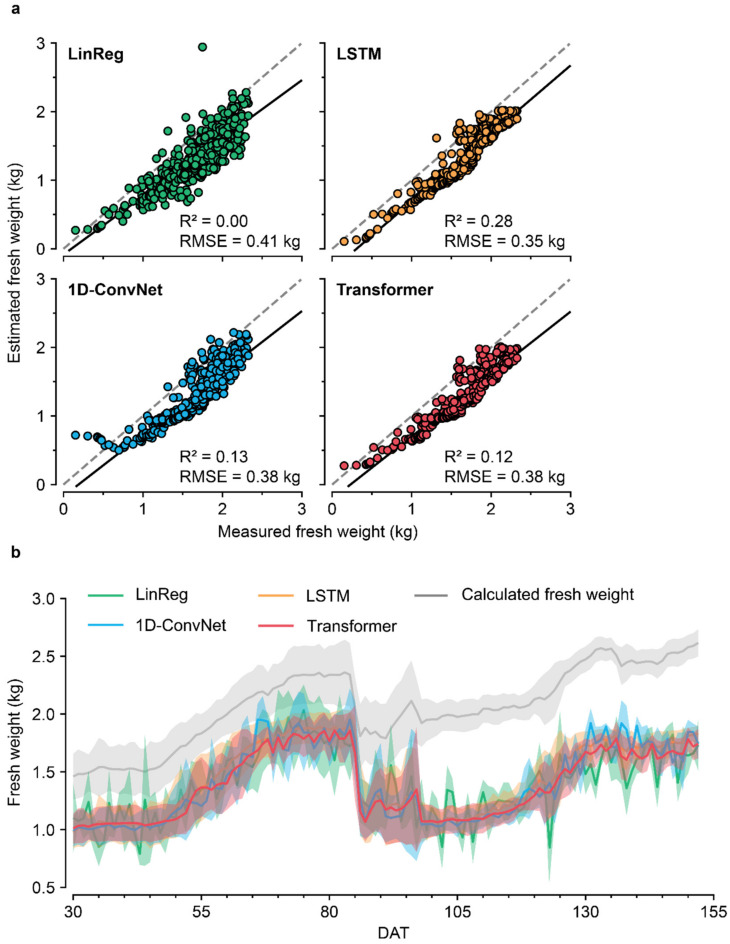
(**a**) Test accuracies of the trained deep learning models for estimating calculated fresh weights from 25 August 2020 to 24 January 2021 (2020W). (**b**) Comparison of the estimations based on days after transplanting (DAT). The standard deviation of the day is represented by a shaded area. LinReg, LSTM, and ConvNet represent linear regression, long short-term memory, and convolution neural network, respectively.

The trained deep learning models showed high validation accuracy (Supplementary Figure S2). For the test data, the models showed low RMSEs and R^2^s (Figure 6a). All the models yielded biased estimations (Figure 6b). The estimations were similar, but the trained Transformer showed a relatively stable output compared with the others. However, the deep learning models showed no advantages compared with the linear regression.

### 3.3. Accuracy of the Estimated Leaf Area

**Figure 7:**
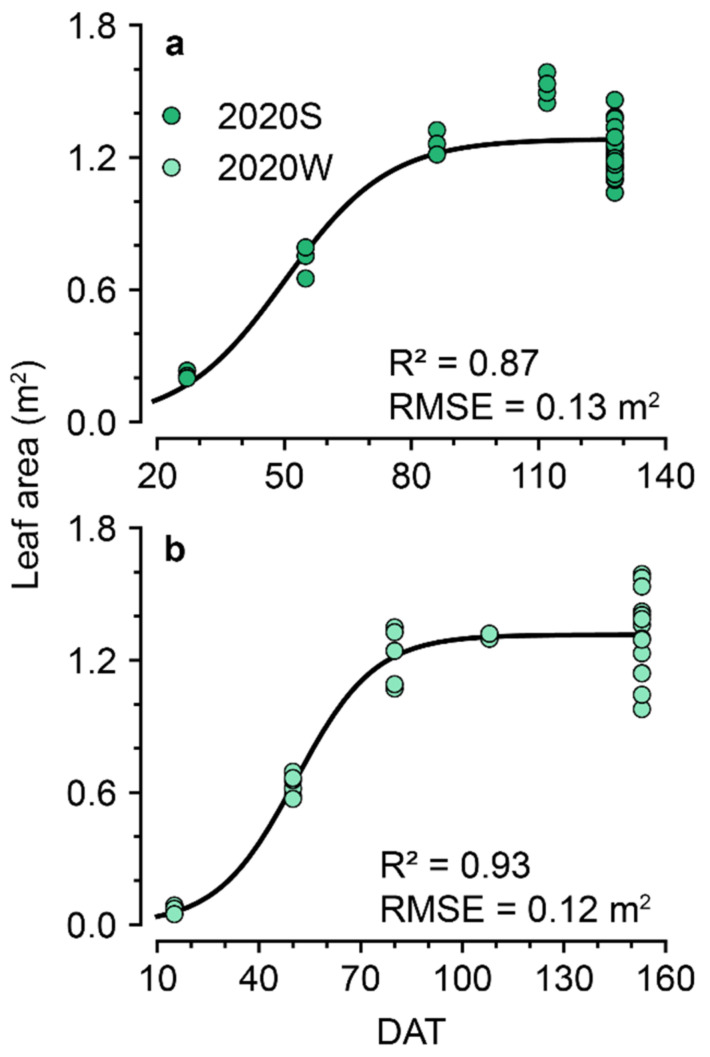
Regressed leaf areas and regression accuracies. The leaf areas from 26 February 2020 to 3 July 2020 (2020S, (**a**)) and from 25 August 2020 to 24 January 2021 (2020W, (**b**)) are depicted. The leaf area was regressed to a sigmoidal function. Refer to Supplementary Table S3 for the fitted coefficients.

Regressed leaf areas in both the cultivation periods recorded approximately R^2^ = 0.9, usable for the labels of ConvNet (Figure 7). Comparing leaf areas in 2020S and 2020W based on increasing DAT, the leaf areas exhibited similar tendencies. However, because the coefficients of the regression for labeling were different, the parameters found in the models were different (Supplementary Table S3). As a model output, the leaf area followed sigmoidal changes, so the regressed leaf area in the latter part of the cultivation exhibited similar values.

**Figure 8:**
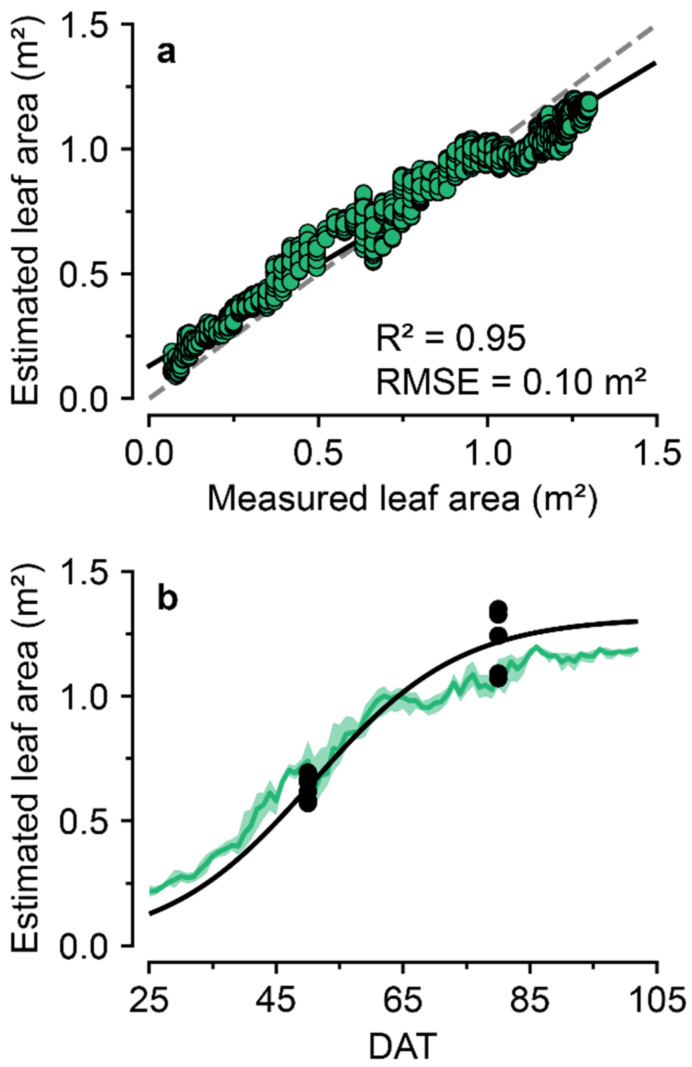
(**a**) Test accuracy of the trained 2D ConvNet for estimating the leaf areas (from 25 August 2020 to 24 January 2021; 2020W). (**b**) Comparison of the estimation based on days after transplanting (DAT). Daily maximum and minimum are depicted by a shaded area.

After the model training, the 2D ConvNet showed unusually high accuracy for the validation datasets (Supplementary Figure S3). However, the model also showed high accuracy for the test datasets (Figure 8). The daily estimations adequately followed their labels, but the model underestimated the leaf areas after 65 DAT. However, the estimation accuracy was high enough that the trained 2D ConvNet seemed to understand the overall growth patterns.

## 4. Discussion

### 4.1. Physiological Comparison of the Two Cultivations

The sweet peppers showed a normal growth pattern, efficiently analyzed using the monitoring system. The leaf areas in 2020S and 2020W showed similar tendencies; however, the cultivar, number of crops per substrate, and planting density were different. In this study, fruit yield was not considered a monitoring factor as the fruit can be heavier than the other organs. Therefore, the fresh weight included unmatured fruit; however, vegetative organs such as stems and leaves accounted for most of the measured fresh weight. Vegetative growth is usually related to abortion of the reproductive organs, and this is tightly managed in greenhouses and hydroponics [37,38]. Since crop management was identically conducted, the management efficiency determined the overall growth pattern. The top of the crops was eliminated in 2020W, thus changing the ratio of vegetative and reproductive organs. Therefore, abortion could explain the decrease in leaf area in 2020W.

### 4.2. Estimated Fresh Weight Using the Simple Calculation

In this study, VWC at night was averaged to calculate the water weight of the substrate (Figure 3). The calculation of the fresh weight reflected the decrease in the total weight caused by the irrigation problem, and it was not too sensitive to VWC changes (Figure 5). The inner water content of crops can determine assimilation efficiency, resulting in changes in fresh and dry weights [39,40].

The method proposed by Lee & Son (2019) was also a simple calculation; however, it could not reflect the tendency of the fresh weight accurately. The difference between the two methods seemed to be due to limitations such as Cf and VWC collection time. Following these two limitations requires a strictly fixed VWC pattern. VWC patterns are affected by crop and sensor conditions [41,42]. Subtracting water weight based on the definition of VWC is more intuitive and requires no parameters. In addition, these limitations could cause overfitting of the given data.

The water content can be kept steady with hydroponics in greenhouses [43]; and hydroponics has a significantly smaller root-zone volume than field culture; that is, the weight of free water is also small compared with the weight of fruits and vegetables [44]. Therefore, a simple calculation based on the definition of VWC and using nighttime values can adequately exclude the water weight in the substrate.

Using the monitoring system and algorithms, the change in fresh weight was monitored. Daily estimation enabled the detection of the decrease in fresh weight, in contrast to the simple regression. The collected data can be used for process-based modeling, usually requiring feedback in specific intervals [45].

### 4.3. Estimated Fresh Weight Using the Trained Deep Learning Models

The deep learning models for estimating crop fresh weight could not overcome biases from the training data (Figure 6). The low RMSE and R^2^ values indicated that the trained deep learning models were highly biased; the tendency of the fresh weight in 2020W was similar to that in 2020S, but the specific weights of water, roots, and substrates were not similar. As all the models were biased similarly, it can be concluded that the model training was successful. A longer period of input data (a week at ten minutes interval) was also not effective (data not shown). The estimation failure seems to result from different semantic distributions of the training and test dataset caused by cultivation difference. In many instances of agricultural research, the amount of data may be insufficient for deep learning algorithms; therefore, the simpler methodology can be helpful in these cases.

Among the models, Transformer showed high performance levels for abstractive tasks [46,47]; and it exhibited stable sequence interpretation for estimating crop fresh weight. However, the model eventually failed to solve the bias problem; in contrast, the simple calculation was successful. For some tasks with small data, a simpler approach could be more applicable than machine learning algorithms that require a large amount of data. Therefore, it is better to apply relatively concise methods first, and deep learning models should be applied for complex tasks.

Additionally, transfer learning was also attempted in this study; however, it was not effective (data not shown). These results suggest that more diverse crops and cultivation data that include similar patterns should be used for the transfer learning [48].

### 4.4. Estimated Leaf Area Using 2D ConvNet

The trained 2D ConvNet showed high accuracy for estimating leaf area. As the validation accuracy was acceptable, the design of the ConvNet structure and the training method was suitable for estimating crop leaf areas. According to the test result of 2020 W, the trained ConvNet accurately predicted the leaf areas from the images. Therefore, the proposed algorithm can support a monitoring system using its characteristic data process.

The ConvNet algorithm was able to relate the images and the crop growth from two different cultivations. The cognitive performance of the abstract target of ConvNet has been reported in several fields, including agriculture [18,27]. Crop images would have diverse information related to crop growth, and ConvNet can generalize this relationship. Therefore, ConvNet can be used to estimate other growth factors; and images can be used for several purposes with deep learning algorithms.

### 4.5. Improvement Potential of the Monitoring Methodology

Fresh weight was overestimated in the early cultivation period to a certain extent. Since VWC did not interrupt the calculation of water weight, it is unlikely that the overestimation resulted from the water weight. Therefore, the overestimation could have been caused by the underestimation of the root weight. Thus, a more accurate measurement method than the oven-drying of the entire substrate is required.

In the latter part of the cultivation, the monitoring system often underestimated target growth factors, possibly due to the structural limitations of the system (Figure 2). The inner frame should be placed between the gutters and the high wires to measure the weight independently. Therefore, the monitoring crops reached the top earlier than the other crops without the system. This space constraint made the final growth of the target crops smaller than the average, also explaining the higher estimation accuracy in 2020W because the top of the crops was eliminated simultaneously. Therefore, the structure of the monitoring system should be improved to avoid interrupting crop growth. However, the overall growth was able to be adequately estimated using the calculation.

For the 2D ConvNet, the monitoring device can be used with high versatility in practice as the average estimation did not fluctuate; however, only the regressed values were used as labels for model training (Figure 7). The 2D ConvNet had to relate the high-variant images with the averaged target values. More cameras and corresponding labels may be the most suitable way to mitigate the problem; however, the cost of installation and data collection may not be realistic. Therefore, deep learning and machine learning models based on probability distribution may be helpful [49,50].

## 5. Conclusions

In this study, the fresh weight and leaf area of sweet peppers were estimated using a simple formula and deep neural networks. A simple calculation using the data from a hanging scale and volumetric water content sensors showed acceptable accuracy for estimating the fresh weight of the crops. The 2D ConvNet accurately estimated the leaf area. The developed methodology could efficiently monitor crop growth under various cultivation conditions. Estimating growth factors can be helpful for crop management, and the collected raw data can be further used to accumulate big data. Unknown factors affecting crop growth can be found in the raw data containing changes in the image and weight. Therefore, a monitoring system that can collect both factors can be widely applied for data analyses, such as machine learning, crop modeling, and data standardization.

## Figures and Tables

**Table 1:** Cultivation and management conditions in different cultivation periods.

Condition	2020S	2020W
Cultivation period	26 February–3 July	26 August–24 January
Planting density	4.08 plants/m^2^	3.06 plants/m^2^
Number of plants	96	84
Cultivar	Scirocco	Mavera and Florate
Topping date	15 June	5 December

**Table 2:** Root and substrate weights collected from destructive investigations from 26 February to 3 July 2020 (2020S) and from 25 August 2020 to 24 January 2021 (2020W). Average values were subtracted from the total weights to calculate crop fresh weights.

Cultivation Period	Root Dry Weight (g/Plant)	Root Dry Weight (g/Slab)	Substrate Weight (g)
2020S	82.98 ± 14.04	297.27 ± 38.81	656.50 ± 30.96
2020W	118.45 ± 23.59	355.37 ± 70.77	887.20 ± 18.74

## Data Availability

Not applicable.

## Supplementary Materials

The following supporting information can be downloaded at: https://www.mdpi.com/article/10.3390/s22207728/s1, Figure S1: Sample images collected from the camera. Images were cropped and resized into 128 × 128, and the resized images were augmented using flipping and shifting; Figure S2: Validation accuracies of the trained deep learning models for estimating the calculated fresh weight.; Figure S3: Validation accuracy of the trained 2D ConvNet for estimating leaf areas; Figure S4: Residual blocks used for the ConvNet model; Table S1: Architectures of deep learning models. LSTM and ConvNet represent a long short-term memory and a convolutional neural network, respectively; Table S2: Parameters used for each model construction and training to estimate the crop fresh weights; Table S3: Regression coefficients for leaf areas in cultivation periods from 26 February 2020 to 3 July 2020 (2020S) and from 25 August 2020 to 24 January 2021 (2020W).
