# Supplementary material for: Non-Destructive Monitoring of Crop Fresh Weight and Leaf Area with a Simple Formula and a Convolutional Neural Network
Source: Sensors (Basel). 2022 Oct 12;22(20):7728. doi: 10.3390/s22207728 (PMC9607460; doi:10.3390/s22207728)
Supplement: Supplementary file 1 [file sensors-22-07728-s001.zip › sensors-1935279-supplementary.pdf]

Table S1. Architectures of deep learning models. LSTM and ConvNet represent a long short-term memory and a convolutional neural network, respectively. Dense is a fully connected layer, a basic form of the neural network. Conv is a convolution layer. Each ConvNet model consisted of convolution layers with the same dimension. Maxpool and Flatten represent the maximum pooling and flattening. Parameters for Conv are denoted as "{type of layer}{kernel size}-{number of filters}," and parameters for the other layers are denoted as "{type of layer}-{number of nodes in the layer}." ResBlock and EncBlock represent a residual block and an encoder block, respectively. The encoder block had a vanilla structure. Refer to Figure S4 for the detailed structure of the residual block. The Conv layers in the latter part of Transformer is ConvNet-like decoder.

Model	LSTM	1D ConvNet	Transformer	2D ConvNet
Input size		144×1		128×128×3
Layers	BiLSTM-64	Conv7-64	Dense-64	Conv7-64
	BiLSTM-64	MaxPool	EncBlock-64	MaxPool
	Dense-32	ResBlock-64	EncBlock-64	ResBlock-64
	Dense-1	ResBlock-128	ResBlock-16	ResBlock-64
		ResBlock-128	ResBlock-32	ResBlock-128
		ResBlock-256	ResBlock-64	ResBlock-128
		ResBlock-512	Conv7-64	ResBlock-128
		Flatten	Conv7-64	ResBlock-256

	Dense-512<br>Dense-128<br>Dense-1	Conv(1,3,5)-1<br>Flatten<br>Dense-1	ResBlock-256<br>ResBlock-512<br>Flatten<br>Dense-512<br>Dense-128<br>Dense-1
Output size		1×1	

Table S2. Parameters used for each model construction and training to estimate the crop fresh weights. Hyphens represent unused values for the corresponding model.

	Value			
Hyperparameter	LSTM	1D ConvNet	Transformer	2D ConvNet
Number of attention heads	-	-	16	-
Embedding dimension	-	-	64	-
Nonlinearity function	Tanh; Sigmoid	ReLU	ReLU, Sigmoid	ReLU
Normalization	Layer	Batch	Batch, Layer	Batch
Batch size	128	128	128	32
Dropout	-	-	0.1	-
Kernel initializer	-	Glorot normal	-	Glorot normal
Padding	-	Same	-	Same
Learning rate	0.002	0.0015	0.004	0.001
Epsilon	1e-08	1e-08	1e-06	1e-06
β_1	0.9	0.9	0.9	0.9
β_2	0.999	0.999	0.999	0.999
Learning rate decay	0.1	0.1	0.1	0.1

Table S3. Regression coefficients for leaf areas in cultivation periods from Feb 26, 2020, to Jul 3, 2020 (2020S) and from Aug 25, 2020, to Jan 24, 2021 (2020W). The data were regressed to a sigmoidal function. Refer to Eq. (1) and Figure 6 for the place of the coefficients and the regression results, respectively.

Cultivation period	Coefficient			
	L	k	x_0	b
2020S	0.01807814296	−0.08168277	2.09198863	0.01408426
2020W	0.00967163573	−0.08633687	6.03753779	0.00735251

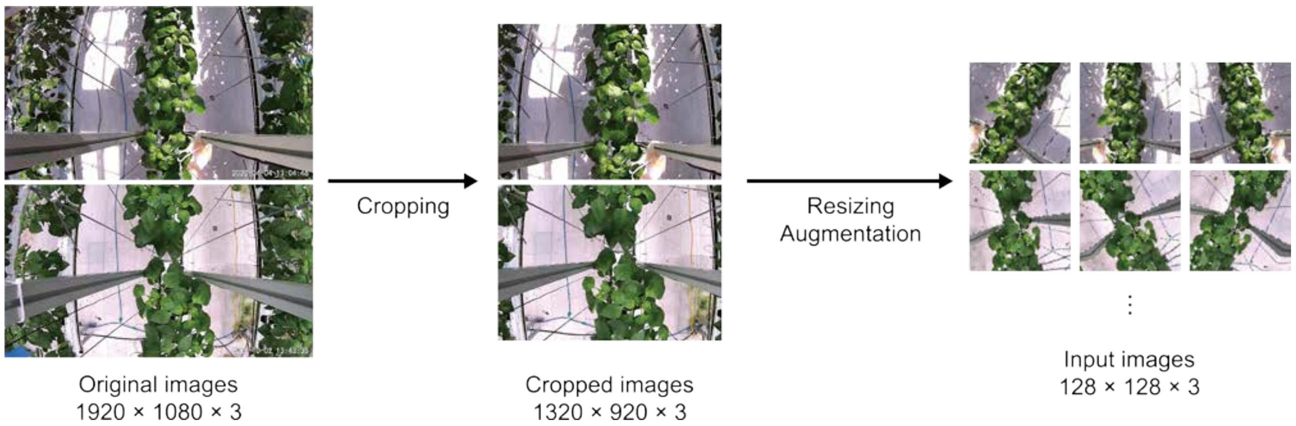

Figure S1. Sample images collected from the camera. Images were cropped and resized into 128 × 128, and the resized images were augmented using flipping and shifting.

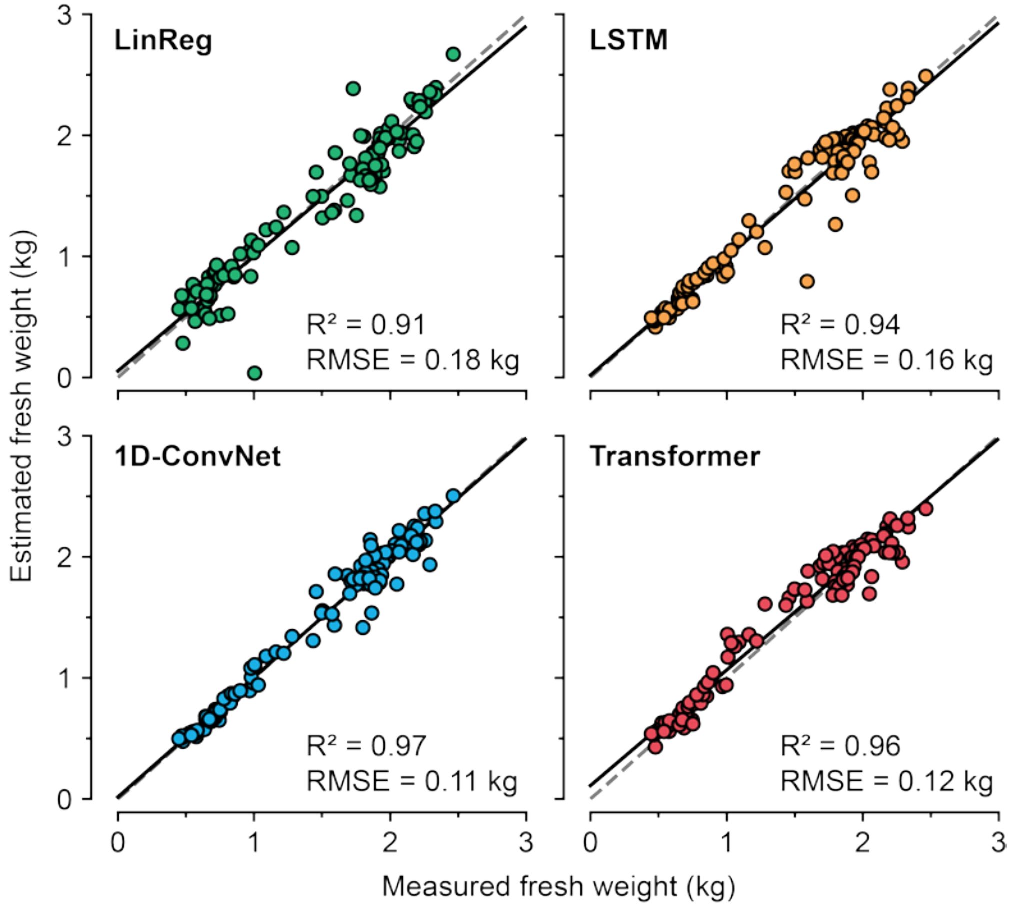

Figure S2. Validation accuracies of the trained deep learning models for estimating the calculated fresh weight. LinReg, LSTM, and ConvNet represent linear regression, long short-term memory, and convolution neural network, respectively. The models were unusually accurate since the tasks were relatively simple. The test accuracy for the cultivation period from Aug 25, 2020, to Jan 24, 2021 (2020W) should be compared for proper evaluation.

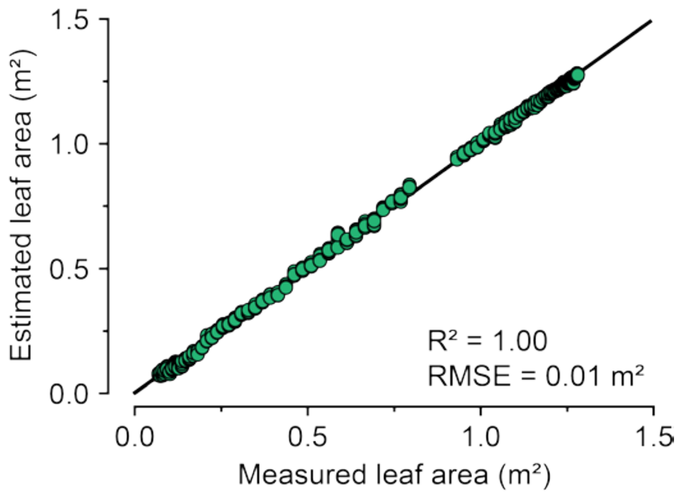

Figure S3. Validation accuracy of the trained 2D ConvNet for estimating leaf areas. The model was unusually accurate since the tasks were relatively simple. The test accuracy for the cultivation period from Aug 25, 2020, to Jan 24, 2021 (2020W) should be compared for proper evaluation of the trained 2D ConvNet.

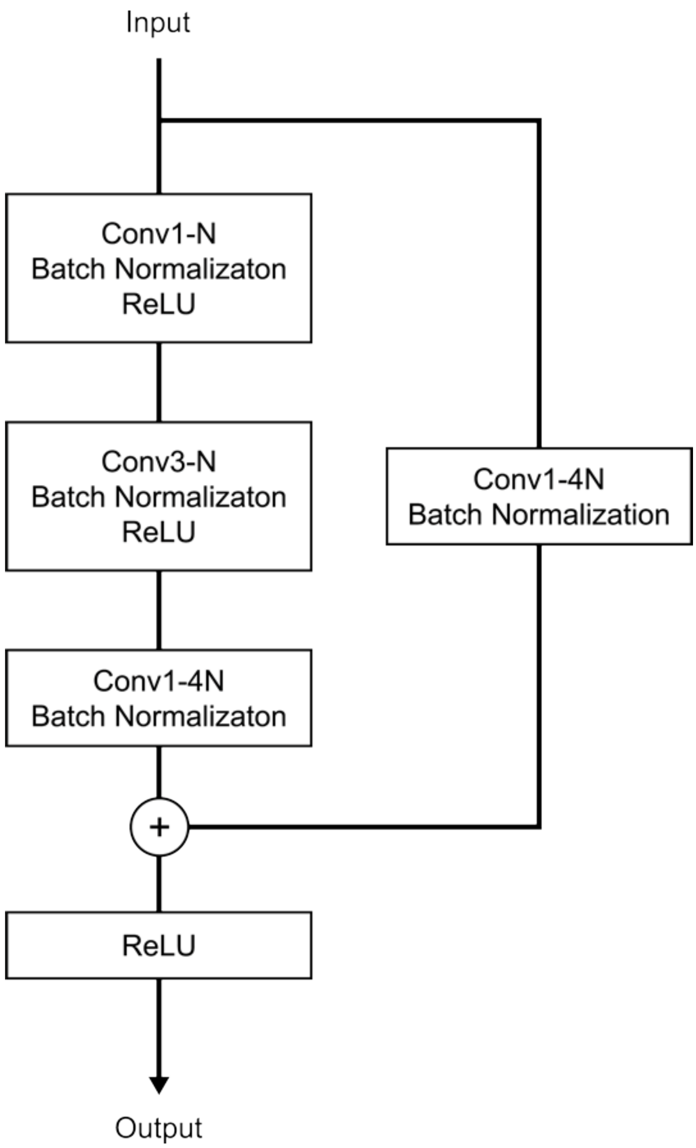

Figure S4. Residual blocks used for the ConvNet model. Parameters for Conv are denoted as "{type of layer}{kernel size}-{number of filters}." N represents a node number that was set previously.
